# A study on blood donor characteristics and preferred environment of South Koreans during the COVID-19 pandemic: A conjoint analysis

**DOI:** 10.3389/fpubh.2023.1138430

**Published:** 2023-03-15

**Authors:** Young-Jae Kim, Jeong-Hyung Cho

**Affiliations:** Department of Physical Education, College of Education, Chunag-Ang University, Seoul, Republic of Korea

**Keywords:** COVID-19, health, blood donation, conjoint analysis, knowledge of blood donation

## Abstract

**Objective:**

The COVID-19 pandemic has caused a decrease in the number of blood donors worldwide, posing a global problem. Therefore, this study investigates people who have continuously participated in blood donation during the COVID-19 pandemic and collects basic information as a reference for maintaining stable amounts of blood during future pandemics.

**Methods:**

The participants of this study were selected from the population of South Korea through stratified sampling considering region and age distributions. Owing to the COVID-19 pandemic, the participants were recruited online through Embrain, an online research and survey company, from June 1 to June 28, 2021. Data were collected from a total of 1,043 participants and used in the study.

**Results:**

The results of this study showed that there was a difference between the donors group and non-donors group in factors such as donation attitude (*F* = 73.342, *p* < 0.001), donation knowledge (*F* = 6.530, *p* < 0.01), and preventive health behavior (*F* = 12.352, *p* < 0.001). Overall, blood donors showed favorable attitude toward and considerable knowledge of blood donation as well as a high level of preventive health behavior. The environment most preferred by people who participated in blood donation during the COVID-19 pandemic was “going with family to a blood donation center that gives out free gifts in a region far away with no confirmed cases,” which showed the highest utility (utility = 0.734).

**Conclusion:**

Even during pandemics, donation attitude, donation knowledge, and preventive health behavior can serve as key factors affecting participation in blood donation. Additionally, blood donation centers that donors can visit with their families represent a favorable environment for the promotion of blood donation during pandemics.

## 1. Introduction

Many people avoid blood donation due to the pain, physical discomfort, psychological fear and side effects (e.g., bruises, vomiting, dizziness) while donating blood (1, 2). Nevertheless, blood is composed of living cells and cannot be preserved for a long time, so continuous blood donation is needed (3). Accordingly, South Korean health organizations have recommended the possession of at least 5 days' worth of blood based on the amount of blood used in the previous year (4). However, anxiety during the COVID-19 pandemic has further reduced the motivation to participate in blood donation (5), and this decline has become a global problem (6, 7).

In general, various factors affect participation in blood donation; however, recently the number of donors has rapidly declined due to the social problems caused by the COVID-19 pandemic (5). According to the World Health Organization (WHO) (8), an average of more than 118.5 million blood donations are collected worldwide in a year; however, the participation rate decreased by 27% due to the COVID-19 pandemic (9, 10). Canada experienced a 22% decline in blood donation (11), and South Korea experienced a 10.4% decline in the number of donors since the outbreak of COVID-19, raising an alert worldwide (12, 13).

Blood shortage puts patients' lives in danger and makes it difficult to cope with national disasters that require large amounts of blood (14). Therefore, it is necessary to investigate people who have continuously participated in blood donation during the COVID-19 pandemic, to collect basic information, that can serve as a reference for maintaining stable amounts of blood in future pandemics.

Clear differences in social changes caused by the COVID-19 pandemic were observed before and after the outbreak. In particular, fear of the new infectious disease caused changes in people's behavior (15). The biggest change in people's behavior due to the COVID-19 pandemic was reflected in preventive health behavior (16). Šurina et al. (17) reported that, to prevent infectious diseases such as COVID-19, people started taking more preventive measures as compared to the past, such as sanitizing their hands, wearing facemasks, and washing their hands when returning home. Notably, people with high levels of preventive behavior engaged more in social activities than those with low levels of preventive behavior (18, 19). This preventive behavior is likely to affect blood donation. For example, even during times like the COVID-19 pandemic, one of the blood donors in the US continuously participated in blood donation until the age of 96 and donated 36 gallons of blood (20), which shows that he had different intentions behind preventive behavior than others.

In fact, previous studies have shown that attitude toward and knowledge of blood donation serve as key factors that affect continuous participation in blood donation (21, 22). Attitude is a key factor in predicting behavior in general (23, 24). In Kim and Yoon (22) and France et al. (25) it was reported that positive attitude toward blood donation affects donation behavior. Moreover, knowledge about blood donation indicates how much one knows about the standards pertaining to blood donation and related behavior, and the level of knowledge about blood donation is one of the key factors affecting donation behavior (26, 27).

Nevertheless, the results of previous studies differed depending with respect to the timing of investigation and the target group (donors and non-donors). The timing of the investigation is particularly important as it is reflective of the current national policy and social conditions. In other words, to obtain reliable indicators for blood donation policies during pandemics, we have to study the relationship among the basic environmental characteristics of blood donors, preventive health behaviors, blood donation attitudes, and knowledge of blood donation during the recent COVID-19 pandemic. Therefore, this study analyzes the characteristics and preferred environment of people who participated in blood donation during the COVID-19 pandemic and compares these participants with the non-donor group and the group of blood donors who donated blood before the COVID-19 pandemic. The objective of this study is to comparatively analyze blood donation attitudes, levels of blood donation knowledge, and preventive health behaviors depending on donation experience during the COVID-19 pandemic and explore the blood donation environment preferred by the group of participants who donated blood during the pandemic. The specific objectives are as follows.

First, this study identifies the differences in blood donation attitudes, donation experiences, and preventive health behaviors depending on the general characteristics of the participants.

Second, this study compares blood donation attitudes, donation experiences, and preventive health behaviors depending on the donation experience of participants during the COVID-19 pandemic.

Third, this study derives the blood donation environment preferred by the group of participants who donated blood during the COVID-19 pandemic.

## 2. Method

### 2.1. Participants

The participants of this study were selected from the population of South Korea through stratified sampling considering region and age distributions. Owing to the COVID-19 pandemic, the participants were recruited online through Embrain, an online research and survey company. The survey was conducted from June 1 to June 28, 2021. Data were collected from a total of 1,043 participants and used in the study. Only those who gave consent before the survey were allowed to participate; the participants were classified into donors and non-donors based on screen items. This study was conducted in compliance with the Declaration of Helsinki and was approved by the Institutional Review Board of Chung-Ang University (1041078-202101-HRSB-023-01).

### 2.2. Measurement tool

The measurement tool used in this study consisted of items that sought information pertaining to attitude toward blood donation, knowledge about blood donation, preventive health behavior, and general characteristics of the participants. The questionnaire consisted of total 41 items, and details of the contents are as follows.

#### 2.2.1. Blood donation attitude

Attitude toward blood donation was evaluated using items that measured perception, emotion, and behavior as potential readiness to participate in blood donation. Perception, which is a subfactor of blood donation attitude, refers to the subjective knowledge or belief that individuals have toward the object; emotion refers to positive or negative feelings toward the object; and behavior refers to the tendency of action toward the object (28). The tool measuring blood donation attitude cited the scale used in Choi's study (29), and consists of a total of 15 questions. The items were measured on a 5-point Likert scale, with higher scores indicating a more positive attitude toward blood donation (“Strongly agree”: 5; “Strongly disagree”: 1). Some examples included “I think blood donation is one of the most rewarding things I can do,” “Blood donation is worth the discomfort,” and “Blood donation is an important duty of citizens.” In Choi's study, the value of Cronbach's α was 0.883 which indicated that the blood donation attitude questionnaire was highly reliable (29), and in this study, the Cronbach's α was 0.856.

#### 2.2.2. Blood donation knowledge

The tool measuring knowledge about blood donation was cited the scale used in Sung's study (30). The tool comprised 14 items, which was assigned score 1 if the participant gave the right answer for each item, and 0 in case of wrong answer. The scores ranged from 0 to 14 points, with higher scores indicating higher level of knowledge about blood donation. Some examples of items are: “I can participate in blood donation until the age of 50,” “People with hepatitis B cannot donate blood,” and “Donating blood makes you lose weight.”

#### 2.2.3. COVID-19 preventive health behavior

COVID-19 preventive health behavior was measured using the tool by Kim & Cho that referred to the infectious disease prevention guide by the United States Centers for Disease Control and Prevention and the basic preventive health behavior guidelines by the Korea Centers for Disease Control and Prevention during the COVID-19 pandemic (19). The tool comprised 11 items rated on a 5-point Likert scale, with higher scores indicating higher level of practice (“Strongly agree”: 5; “Strongly disagree”: 1). Some examples items are: “I avoid visiting crowded places,” “I wear a mask when I have respiratory symptoms such as fever or cough,” and “I ventilate constantly to maintain fresh air inside.” In Kim and Cho's study, the Cronbach's α value for reliability of the blood donation attitude questionnaire was 0.838 (19), and in this study, it was 0.755.

### 2.3. Data analysis

All analyses in this study were conducted using SPSS version 26.0 through coding and data cleaning processes. Frequency and descriptive analyses were conducted for sociodemographic characteristics, and Cronbach's α was used to ensure the reliability of the tool. Additionally, two-way ANOVA was conducted to compare the differences between groups, and conjoint analysis was conducted to determine the environment preferred by the blood donors.

## 3. Results

### 3.1. Participant characteristics

In this study, the participants were divided into three groups according to their general characteristics and participation in blood donation. Specific details are shown in Table 1. First, there were *n* = 549 female participants (51.9%), which were more than male participants, and the average age of participants was 40.6 years. There were more married participants (*n* = 530, 50.8%) than singles, and most of the participants (*n* = 520, 49.9%) stated that their health status was average. By comparing the groups that participated in blood donation before and after the COVID-19 pandemic, it was found that the participants in the latter group were mostly young (average age = 33.6 years), male (52.4%), single (61.1%), and those who were relatively confident about their health (56.3%). Those in the former group were mostly middle-aged (average age = 44 years), females comprised (51.1%), and married (62.7%) of the total sample.

**Table 1:** Participant characteristics.

**Item**	**Total response**	**Non -participant**	**Participated before the COVID-19 pandemic**	**Participated after the COVID-19 pandemic**
	***N*** = **1,0443 (%)**	***N*** = **508 (%)**	***N*** = **327 (%)**	***N*** = **208 (%)**
**Gender**				
Male	503 (48.1)	234 (46.1)	160 (48.9)	109 (52.4)
Female	540 (51.9)	274 (53.9)	167 (51.1)	99 (47.6)
**Age**				
Mean (SD)	40.6 (15.8)	40.2 (16.2)	44.4 (13.3)>	33.6 (16.1)
Range	15–65			
**Marital status**				
Single	513 (49.2)	264 (52.0)	122 (37.3)	127 (61.1)
Married	530 (50.8)	244 (48.0)	205 (62.7)	81 (38.9)
**Subjective health**				
Good	398 (38.2)	170 (33.5)	111 (33.9)	117 (56.3)
Natural	520 (49.9)	257 (50.6)	187 (57.2)	76 (36.5)
Bad	125 (12.0)	81 (15.9)	29 (8.9)	15 (7.2)

*N*, number, SD, standard deviation.

### 3.2. Analysis of differences in attitudes, knowledge, and behaviors between donors and non-donors

Table 2 shows the differences in “donation attitude,” “donation knowledge,” and “preventive health behavior” between the donors (*n* = 535) and non-donors (*n* = 508). The findings demonstrated differences in donation attitudes (*F* = 73.342, *p* < 0.001), donation knowledge (*F* = 6.530, *p* < 0.01), and preventive health behaviors (*F* = 12.352, *p* < 0.001). Overall, blood donors showed positive attitude as well as high level of knowledge and preventive health behavior.

**Table 2:** Analysis of differences in attitude, knowledge, and preventive health behavior of blood donors.

**Item**		**Total (M)**	**Gender**		**Age**		**Marital status**		**Subjective health**		
			**Male**	**Female**	**Young (30 or less)**	**Old (31 or more)**	**Single**	**Married**	**Good**	**Natural**	**Bed**
Attitude	Pa	535 (4.0)	269 (4.0)	266 (3.9)	259 (3.9)	276 (4.0)	249 (3.9)	286 (4.0)	228 (4.1)	263 (3.9)	41 (3.9)
	Np	508 (3.7)	234 (3.6)	274 (3.7)	246 (3.7)	266 (3.7)	264 (3.7)	244 (3.7)	170 (3.8)	257 (3.6)	81 (3.6)
Total	*F*	73.342	0.014		0.329		3.536		16.138		
	*p*	0.000	0.905		0.567		0.060		0.000		
Knowledge	Pa	535 (9.3)	269 (9.2)	266 (9.5)	259 (9.3)	276 (9.4)	249 (9.2)	286 (9.5)	228 (9.2)	263 (9.4)	44 (9.7)
	Np	508 (9.1)	234 (9.0)	274 (9.1)	242 (8.8)	266 (9.3)	264 (8.8)	244 (9.3)	170 (8.9)	257 (9.1)	81 (9.1)
Total	*F*	6.530	3.580		6.995		12.862		2.727		
	*p*	0.011	0.059		0.008		0.000		0.066		
Preventive Health Behavior	Pa	540 (4.1)	269 (4.0)	266 (4.1)	259 (4.0)	276 (4.1)	249 (4.0)	286 (4.2)	228 (4.2)	263 (4.0)	44 (3.9)
	Np	503 (4.0)	234 (3.9)	274 (4.0)	242 (3.9)	266 (4.0)	264 (3.9)	244 (4.0)	170 (4.1)	257 (4.0)	81 (3.8)
Total	*F*	12.352	15.192		10.399		18.575		16.029		
	*p*	0.000	0.000		0.001		0.000		0.000		

Pa, participant; Np, non-participant; *F, F*-ratio; *P, P*-value; M, mean.

### 3.3. Analysis of differences in attitude, knowledge, and behavior between donors before and donors after the outbreak of COVID-19

Table 3 shows the differences in “donation attitudes,” “donation knowledge,” and “preventive health behaviors” between participants who donated blood before the outbreak of COVID-19 (*n* = 327), and those who donated blood after the outbreak of COVID-19 (*n* = 208). Specifically, those who participated in blood donation after the outbreak of COVID-19 showed a statistically significant difference in donation attitudes (*F* = 33.882, *p* < 0.001).

**Table 3:** Analysis of differences in attitude, knowledge, and preventive health behavior according to participation in blood donation during the COVID-19 pandemic.

**Item**	**Total (M)**		**Gender**		**Age**		**Marriage**		**Subjective health**		
			**Male**	**Female**	**Young (30 or less)**	**Old (31 or more)**	**Single**	**Married**	**Good**	**Natural**	**Bed**
Blood donation attitude	Af	208 (4.1)	109 (4.2)	99 (4.0)	137 (4.1)	74 (4.1)	127 (4.1)	81 (4.1)	117 (4.2)	76 (4.0)	15 (3.7)
	Be	327 (3.9)	160 (3.8)	167 (3.9)	125 (3.8)	202 (3.9)	122 (3.7)	205 (3.9)	111 (3.9)	187 (3.8)	29 (4.0)
Total	*F*	33.882	3.932		3.762		5.890		4.978		
	*p*	0.000	0.048		0.053		0.016		0.007		
Blood donation knowledge	Af	208 (9.2)	109 (9.0)	99 (9.3)	137 (9.2)	74 (9.2)	127 (9.1)	81 (9.2)	117 (9.0)	76 (9.4)	15 (9.2)
	Be	327 (9.4)	160 (9.3)	167 (9.5)	125 (9.4)	202 (9.5)	122 (9.3)	205 (9.5)	111 (9.4)	187 (9.4)	29 (10.0)
Total	*F*	2.993	0.024		0.010		0.001		1.012		
	*p*	0.084	0.876		0.922		0.979		0.364		
Preventive health behavior	Af	208 (4.1)	109 (4.1)	99 (4.1)	137 (4.1)	74 (4.2)	127 (4.1)	81 (4.2)	117 (4.2)	76 (4.1)	15 (3.7)
	Be	327 (4.0)	160 (3.9)	167 (4.2)	125 (4.0)	202 (4.1)	122 (3.9)	205 (4.1)	111 (4.1)	187 (4.0)	29 (4.0)
Total	*F*	2.806	9.813		0.014		0.845		3.740		
	*p*	0.095	0.002		0.906		0.358		0.024		

Af, participant after the COVID-19 pandemic; Be, participant before the COVID-19 pandemic.

The interesting result is that the factors of attitude and behavior were higher in those who participated in blood donation during the COVID-19 pandemic, whereas knowledge was higher in those who participated before the outbreak of COVID-19 and did not participate during the pandemic (*F* = 2.993, *p* < 0.08). This indicates that people with better knowledge are more cautious about donating blood in a pandemic.

### 3.4. The preferred environment of blood donors during the COVID-19 pandemic

Conjoint analysis combines the attributes that are considered important by participants among various social factors, which facilitate the identification of the partial value of each attribute, calculating the fitness, and developing a new model preferred by the participants based on the ideal and optimal combination (31). Following (32), the independent variable (blood donation environment) affects the dependent variable (participation in blood donation) through the mechanism of additive composition, the following equation was obtained:


(1) $$\begin{array}{l} V_{\left(x\right)}=A_{\left(a\right)}+B_{\left(b\right)}+C_{\left(c\right)}+D_{\left(d\right)} \end{array}$$

In this equation, participation V(x) comprises effects of the A(a), B(a), the C(a), and D(a) factors, indicating the sum of utilities obtained from each attribute.

Accordingly, conjoint analysis was conducted to analyze the environment preferred by blood donors during the COVID-19 pandemic. Conjoint analysis was conducted in the order of setting the attributes and their levels, constructing the conjoint design, and deriving the key preferred attributes. Specific results are as follows.

Table 4 shows the key attributes of blood donors and the attribute levels. Based on the preliminary survey and expert advice, the key attributes of blood donors are “region,” “place,” “companion,” and “free gifts” (33–36). For the region attribute, there were two attribute levels: a region nearby where there were confirmed cases of COVID-19, and a region far away where there were no confirmed cases of COVID-19. For the place attribute, there were three levels: hospital, blood donation center, and mobile blood donation vehicle. For the companion attribute, there were three levels: individual, friend or colleague, and family. Finally, for the free gifts attribute, there were two levels: necessary and not necessary. Each of the attributes and their levels considered in this study are shown in Table 4.

**Table 4:** Attributes and attribute levels of the blood donation environment.

**Attributes**	**Attribute level**
Region	(1) A place that is nearby but has confirmed cases of COVID-19, (2) a place that is far away but has no confirmed cases of COVID-19
Place	(1) Hospital, (2) blood donation center, (3) mobile blood donation vehicle
Companion	(1) Individual, (2) friend or colleague, (3) family
Free gifts	(1) Necessary, (2) does not matter

Table 5 presents cards for the survey using the orthogonal plan of SPSS Software, comprised of nine cards.

**Table 5:** Blood donation environment factor design.

**Card**	**Region**	**Place**	**Companion**	**Free gifts**
1	A place nearby with confirmed cases	Blood donation center	Alone	Does not matter.
2	A place far away with no confirmed cases	Hospital	Friend or colleague	Does not matter.
3	A place nearby with confirmed cases	Blood donation center	Friend or colleague	Necessary.
4	A place nearby with confirmed cases	Mobile blood donation vehicle	Family	Does not matter.
5	A place far away with no confirmed cases	Blood donation center	Family	Necessary.
6	A place nearby with confirmed cases	Mobile blood donation vehicle	Friend or colleague	Necessary.
7	A place nearby with confirmed cases	Hospital	Alone	Necessary.
8	A place far away with no confirmed cases	Mobile blood donation vehicle	Alone	Necessary.
9	A place nearby with confirmed cases	Hospital	Family	Necessary.

The results of conjoint analysis on the environment preferred by blood donors during the COVID-19 pandemic are shown in Table 6. Specifically, “place” was the factor considered most important by blood donors, showing 37.6% of relative importance out of 100%, followed by “companion” (27.1%), “region” (20.2%), and “free gifts” (14.9%). As for utility, for the place attribute, “blood donation center” had a high utility of 0.215. “Family” had a utility of 0.160 in the “companion' attribute”, and “a place far away with no confirmed cases” had a utility of 0.143 in the “region” attribute. Finally, “necessary” had the highest utility at 0.216 in the “free gifts” attribute. The conjoint model was adequate with Pearson's *R* = 4.976, *p* < 0.027.

**Table 6:** Conjoint analysis results for the blood donation environment.

	**Factor**	**Utility**	**S. E**	**Importance (%)**
Region	A place nearby with confirmed cases	−0.143	0.299	20.248
	A place far away with no confirmed cases	0.143	0.299	
Place	Hospital	0.024	0.399	37.656
	Blood donation center	0.215	0.399	
	Mobile blood donation vehicle	−0.239	0.399	
Companion	Alone	0.071	0.399	27.107
	Friend	−0.231	0.399	
	Family	0.160	0.399	
Free gifts	Necessary	0.216	0.299	14.989
	Not necessary	−0.216	0.299	
Constant		4.976	0.315	
Pearson's *R*		0.659	*P*	0.027

S. E, standard error.

Table 7 shows the total utility of the profiles of blood donation environment preferred by people who participated in blood donation during the COVID-19 pandemic. Overall, the profile of “going with family to a blood donation center that gives out free gifts in a region far away with no confirmed cases” showed the highest utility (Utility = 0.734). This can be used as a reference for creating an environment that promotes the continuous participation in blood donation and for recruiting new blood donors.

**Table 7:** Profile utility of the environment preferred by blood donors.

**Card**	**Region**	**Place**	**Companion**	**Free gifts**	**Utility**	**Rank**
1	A place nearby with confirmed cases	Blood donation center	Alone	Does not matter.	−0.073	6
P.U	−0.143	0.215	0.071	−0.216		
2	A place far away with no confirmed cases	Hospital	Friend or colleague	Does not matter.	−0.280	7
P.U	0.143	0.024	−0.231	−0.216		
3	A place nearby with confirmed cases	Blood donation center	Friend or colleague	Necessary.	0.057	5
P.U	−0.143	0.215	−0.231	0.216		
4	A place nearby with confirmed cases	Mobile blood donation vehicle	Family	Does not matter.	−0.438	9
P.U	−0.143	−0.239	0.160	−0.216		
5	A place far away with no confirmed cases	Blood donation center	Family	Necessary.	0.734	1
P.U	0.143	0.215	0.160	0.216		
6	A place nearby with confirmed cases	Mobile blood donation vehicle	Friend or colleague	Necessary.	−0.397	8
P.U	−0.143	−0.239	−0.231	0.216		
7	A place nearby with confirmed cases	Hospital	Alone	Necessary.	0.168	4
P.U	−0.143	0.024	0.071	0.216		
8	A place far away with no confirmed cases	Mobile blood donation vehicle	Alone	Necessary.	0.191	3
P.U	0.143	−0.239	0.071	0.216		
9	A place nearby with confirmed cases	Hospital	Family	Necessary.	0.257	2
P.U	−0.143	0.024	0.160	0.216		

P.U, part utility.

## 4. Discussion

This study analyzed the characteristics of those who participated in blood donation during the COVID-19 pandemic to obtain information that can increase their participation during pandemics. Specifically, it comparatively analyzed the characteristics of two donor groups: one without donation experience while the other that donated blood only before the outbreak of COVID-19. Moreover, this study found the blood donation environment that was preferred by those who participated in blood donation during the COVID-19 pandemic, thus providing valuable information to promote blood donation during infectious disease.

First, the comparison of donors and non-donors revealed that all factors pertaining to attitude, knowledge, and preventive health behavior were high in those with donation experience. This is consistent with the results of previous studies, indicating that attitude, knowledge, and preventive health behavior are closely related to participation in blood donation even during a pandemic (37–39).

Volunteering behavior such as blood donation was found to affect participation depending on the attitude, with more positive attitudes resulting in increased participation in blood donation (21). This study also revealed that those who participated in blood donation during the COVID-19 pandemic showed increased positive attitude than those who did not, proving that, despite the decrease in blood donors due to the pandemic, attitude toward donation still served as a key factor affecting participation. Moreover, since blood donors showed statistically high scores in all social characteristics during the COVID-19 pandemic, attitude toward blood donation proved to be a key variable that increased the rate of participation in blood donation during pandemics.

Further, knowledge of blood donation was identified as a factor that directly affected participation in blood donation, which varied depending on the social environment. In (40), donation knowledge in developing countries was examined, to find out the differences among countries. The current knowledge of blood donation of South Koreans differed as compared to the past when it was a developing country. A study on blood donation knowledge in the past showed that the blood donation knowledge of participants (rate of correct answers) was 46.8% (41). Meanwhile, more recently, according to (42), the rate increased to 76.4%. However, the current study showed a decrease to 66.4%. Notably, those who participated in blood donation during the COVID-19 pandemic scored relatively lower than those who donated blood before it. The results of this study showed that those who donated blood during the pandemic had generally low levels of knowledge about blood donation, but they showed high rates of correct answers for items related to illnesses, such as “People with hepatitis B cannot donate blood” (89% correct answers among participants after COVID-19 vs. 83% before COVID-19) and “You cannot donate blood after taking medicine” (88% correct answers among participants after COVID-19 vs. 81% before COVID-19). This shows that people with a high level of understanding about blood donation related to illness are more enthusiastic about donating blood. Therefore, when launching campaigns to promote blood donation during a pandemic, it will be effective to include information about blood donation in situations related to the pandemic.

Preventive health behavior is important in pandemics such as the COVID-19 (43), which also applies to blood donation. To supply blood during a pandemic, it is necessary to increase donors' compliance with public health (6). This is because COVID-19 may spread through blood donation. COVID-19 is a contagious disease that can cause damage by becoming a host and spreading to others (44). Interestingly, there was a difference among blood donors according to gender, with men showing a higher rate of blood donation during the COVID-19 pandemic, while women showed a lower rate. This could be because the virus affects men and women differently (45). In the United States, twice more male deaths from COVID-19 were reported as compared to female deaths, and in South Korea also number of male deaths was more than female deaths. In other words, as COVID-19 persists, men are more sensitive to preventive health behavior.

Finally, as a result of conjoint analysis to identify the environment preferred by blood donors during the COVID-19 pandemic, place was considered the most important factor. Interestingly, those who donated blood before the COVID-19 pandemic preferred hospitals, whereas those who donated blood after the COVID-19 pandemic preferred blood donation centers. According to a survey by (46), effective participation occurred when hospital doctors and nurses encouraged blood donation. This indicates the level of people's trust and their reliance on hospitals, including those in South Korea. The overseas expansion of South Korean medical institutions is three times higher than that of China and the United States, and the number of foreigners coming to South Korea for medical treatment had exceeded 260,000 (47). Moreover, the average life expectancy of South Koreans is higher than the OECD average, indicating high reliability on hospitals. However, since about 50% of people have avoided visiting hospitals after the outbreak of COVID-19 (48), those who participated in blood donation during the COVID-19 pandemic preferred blood donation centers, as an institution specializing in blood donation, over hospitals. Interestingly, blood donors preferred to visit with their families over visiting alone when donating blood. This is consistent with the previous results that family support during the COVID-19 pandemic is helpful for mental health in overcoming everyday difficulties and depression (49, 50). Moreover, COVID-19 is more likely to be transmitted by strangers, but families are more trustable because they spend most of the time together. Compiling the profile utilities of the environment preferred by blood donors during the COVID-19 pandemic, the most preferred environment was “going with family to a blood donation center that gives out free gifts in a region with no confirmed cases”. Therefore, to establish measures that can increase blood donors, it will be effective to come up with a promotional strategy based on the findings of this study.

### 4.1. Strengths and limitations

This study provided important information to increase participation in blood donation during a pandemic such as the COVID-19 pandemic by analyzing the characteristics of people who actually participated in blood donation during the COVID-19 pandemic and ascertaining the environment preferred by them. Nonetheless, this study had the following limitations. Since this study was conducted on people who participated in blood donation during the COVID-19 pandemic, the results must not be interpreted equally in situations other than pandemics. Moreover, since each country may have a different culture or environment for blood donation, the results must be interpreted according to the individual situation of each country. In addition, the results of the difference analysis section (Tables 2, 3) of this study are statistically significant, but they may appear differently in the actual environment because the observed values are small.

## Data availability statement

The raw data supporting the conclusions of this article will be made available by the authors, without undue reservation.

## Ethics statement

The studies involving human participants were reviewed and approved by Institutional Review Board at Chung-Ang University (1041078-202101-HRSB-023-01). The patients/participants provided their written informed consent to participate in this study.

## Author contributions

Y-JK and J-HC: conceptualization, methodology, validation, and review and editing. J-HC: formal analysis and data curation. Y-JK: investigation and writing original draft. All authors were involved in writing the manuscript and approved its final version.
